# SEOM clinical guidelines for the treatment of head and neck cancer (2020)

**DOI:** 10.1007/s12094-020-02533-1

**Published:** 2021-02-26

**Authors:** R. Mesia, L. Iglesias, J. Lambea, J. Martínez-Trufero, A. Soria, M. Taberna, J. Trigo, M. Chaves, A. García-Castaño, J. Cruz

**Affiliations:** 1 Institut Català d’Oncologia, Badalona, Spain; 2 Servicio de Oncología Médica, Hospital Universitario 12 de Octubre, 12 de Octubre, Madrid, Spain; 3 Servicio de Oncología Médica, Hospital Clínico Universitario Lozano Blesa, Zaragoza, Spain; 4 Servicio de Oncología Médica, Hospital Universitario Miguel Servet, Zaragoza, Spain; 5 Servicio de Oncología Médica, Hospital Universitario Ramón y Cajal, Madrid, Spain; 6 Servicio de Oncología Médica, Hospital Clínico Virgen de la Victoria, Málaga, Spain; 7 Servicio de Oncología Médica, Hospital Virgen de Valme, Seville, Spain; 8 Servicio de Oncología Médica, Hospital Universitario Marqués de Valdecilla, Santander, Spain; 9 Servicio de Oncología Médica, Hospital Clínico Universitario de Salamanca, Salamanca, Spain; 10 Institut Català d’Oncologia, Hospitalet de Llobregat, Spain

**Keywords:** Immunotherapy, Human papillomavirus, Head and neck cancer, Chemoradiotherapy

## Abstract

Head and neck cancers (HNC) are defined as malignant tumours located in the upper aerodigestive tract and represents 5% of oncologic cases in adults in Spain. More than 90% of these tumours have squamous histology. In an effort to incorporate evidence obtained since 2017 publication, the Spanish Society of Medical Oncology (SEOM) presents an update of the squamous cell HNC diagnosis and treatment guideline. Most relevant diagnostic and therapeutic changes from the last guideline have been updated: introduction of sentinel node biopsy in early oral/oropharyngeal cancer treated with surgery, concomitant radiotherapy with weekly cisplatin 40 mg/m^2^ in the adjuvant setting, new approaches for HPV-related oropharyngeal cancer and new treatments with immune-checkpoint inhibitors in recurrent/metastatic disease.

**Supplementary Information:**

The online version contains supplementary material available at 10.1007/s12094-020-02533-1.

## Introduction

Squamous cell carcinomas of the head and neck (SCCHN) are defined as malignant tumors arising from mucosal surfaces located in the upper aerodigestive tract (paranasal sinuses, nasopharynx, oropharynx, hypopharynx, larynx, oral cavity, nostrils). In Spain, SCCHN represents 5% of all new cancer diagnoses in adults, being the sixth neoplasm (fifth in men), similar to the European median, and a mortality rate of three points below compared to the European median [1]. The most important risk factor in our region continues to be tobacco and alcohol use, but human papillomavirus (HPV) infection is a key etiological factor in oropharyngeal cancer burden, which is rising worldwide [2]. Despite the majority patients with early-stage SCCHN can be cured with surgery or radiation, those with aggressive disease and those with locally advanced stages, that represents two-thirds of new diagnosis, are more likely to recur (50% 5-year overall survival) [2]. A multidisciplinary team, bringing together all professionals who specialize in the diagnosis and treatment of these patients, will make the decision to establish the best sequence of individualized treatment for each patient. Within what is known as SCCHN, each location has a clinical presentation, staging, prognosis and different therapeutic approach. As this is a general guide, the particularities of each subsite will not be dealt with in depth. Nasopharyngeal cancer with a different epidemiological, pathological and natural history will not be included in this guide.

## Methodology

SEOM guidelines have been developed with the consensus of ten oncologists from the Spanish Group for the Treatment of Head and Neck Tumors (TTCC) and SEOM. To assign a level and quality of evidence and a grade of recommendation to the different statements of this treatment guideline, the Infectious Diseases Society of America-US Public Health Service Grading System for Ranking Recommendations in Clinical Guidelines was used (Table 1). The final text has been reviewed and approved by all authors.

**Table 1:** Strength of recommendation and quality of evidence score

Category, grade	Definition
Strength of recommendation	
A	Good evidence to support a recommendation for use
B	Moderate evidence to support a recommendation for use
C	Poor evidence to support a recommendation
D	Moderate evidence to support a recommendation against use
E	Good evidence to support a recommendation against use
Quality of evidence	
I	Evidence from ≥ 1 properly randomized, controlled trial
II	Evidence from ≥ 1 well-designed clinical trial, without randomization; from cohort or case controlled analytic studies (preferably from > 1 centr*e); from multiple time series; or from dramatic results from uncontrolled experiments
III	Evidence from opinions of respected authorities, based on clinical experience, descriptive studies, or reports of experts committees

*Studies reported as abstracts in congresses pending journal publication at the time of writing this guide

## Diagnosis and staging

It is essential to start the diagnostic process with a good clinical history, including toxic and sexual habits and a methodical physical examination, with special attention to the head and neck area (inspection, indirect mirror examination or direct endoscopy and palpation of primary sites and neck).

To explore tumor extension, diagnosis imaging is needed:

-Imaging diagnosis before a large biopsy avoids false diagnosis from anatomy distortion [2].
-Cervical computed tomography (CT) or magnetic resonance (MR). Imaging MRI is superior to CT for evaluation of tongue, perineural spread, skull base invasion and intracranial extension. Regarding lymphatic dissemination, defining extracapsular nodal extension is of prognostic value.
-CT of chest preferably, or X-ray in early stages.
-Positron emission tomography-CT (PET-CT) is very useful in diagnosis of node (N) and metastases (M) and synchronous primary tumors. It is recommended in patients with stage III–IV disease when definitive treatment is indicated or in those with equivocal findings on CT or MRI scan [3].
-Esophageal–gastric contrast study or esophagoscopy in case of dysphagia.
-Histological diagnosis is mandatory by primary tumor biopsy or fine needle aspiration (FNA) of lymph nodes (biopsy is always better than FNA). If a node biopsy is needed, complete nodal resection is preferable to prevent extracapsular metastatic spread [2].
-Functionalism evaluation: chewing, swallowing, phonation, breathing (stability of the airway mast be assessed), odontology and nutritional status.
-Special evaluations if needed: psychological and social situation, cessation of smoking or alcohol dependence.

Accurate staging is crucial for coordinating and tailoring therapy to each individual patient. The 8th edition of TNM classisfication was implemented from January, 2018: [4]

-The most important introduction is an independent classification for p16-positive oropharyngeal tumors: in the T category, T4a and T4b were pooled as T4, and N category was reclassified. As a consequence, there is a downstaging.
-T category (T1–T3) of lip and oral cavity includes the extent of depth invasion.
-N3 category for non-HPV related tumors has been subdivided into N3a and N3b according to extranodal extension (in N1 and N2 categories lack of extranodal extension is specified).
-Perineural invasion or deep invasion is included within squamous cell carcinomas of the skin.

## Early disease (clinical stage I–II) treatment

Surgery, 3D conformal radiotherapy (RT) and brachytherapy provide similar locoregional control and survival outcomes, but they have not been compared in randomized trials [2]. A multidisciplinary team should choose according to the characteristics and wishes of the patient and the potential functional outcomes, a single modality to avoid morbidity.

Transoral resection is preferred over RT in oral cavity because of the decreased long-term morbidity (II, B) [5]. In oropharyngeal carcinoma, minimally invasive transoral surgery such as robot (TORS) or laser (TLM), in selected patients, should be prioritized over open surgery (II, B) [6]. Alternative RT seems to have less tendency to long-term dysphagia (II, B) [7].

In both locations, cervical lymph nodes should be treated with prophylactic radiation or elective neck dissection (bilaterally in tumors that arise in or near the midline and guided by location and depth in oral carcinoma) (II, B) [8].

Recent data recommend treatment based on sentinel node biopsy for oral cavity and oropharynx tumors (T1–2 N0), since it obtains the same neck-relapse-free survival at 2 years than the neck dissection, with less morbidity during first year post-surgery (I, A) [9]. When cervical dissection is indicated, we would recommend elective neck dissection over therapeutic neck dissection due to similar efficacy with less morbidity associated (I,A) [10]. In the choice of treatment for hypopharyx and larynx carcinomas, laryngeal functional results will be considered in addition to survival. Conservative laryngeal surgery (TLM or supraglottic or supracricoid laryngectomy) will be priorized over open surgery, and considerer RT treatment in case of requiring extensive surgical resection [11, 12]. Elective treatment of the neck in hypopahrynx and supraglottic cancer is recommended (II, B), but not in glottic neoplasm (III, C).

If the pathological staging is superior to the clinical staging or there are poor prognosis factors, complementary treatments should be used (I, A) including the re-resection.

## Locally advanced disease (clinical stages III, IVA, IVB) treatment

In all cases there must be a multidisciplinary assessment to decide the best combined treatment option for each patient either based on surgery or RT as the key treatment (I-A) Given its special interest, we will introduce a special section for larynx preservation and HPV-related oropharyngeal cancer treatments.

### Surgery-based treatment

There is no universally accepted definition of unresectability in SCCHN, but some anatomical criteria are considered unequivocal and classified as T4b tumors (involvement of skull base, cervical vertebrae, prevertebral muscles, brachial plexus, mediastinal spread, involvement of nasopharynx, fixed tumor to collarbone, vascular encasement) [13].

Multidisciplinary Tumor Boards can exclude patients for surgery: few chances of achieving adequate margins, unacceptable functional and/or esthetic sequelae, little expectation of cure or due to patients’ comorbidities.

For patients with T3-4aN0 tumors an ipsilateral or bilateral neck dissection is an option (except oral cavity where it is mandatory). When neck nodes are palpable, all nodal levels should be dissected [14].

#### Adjuvant treatment

-Preferred Radiation technique: Intensity Modulated Radiotherapy (IMRT) 60–66 Gy (I-A) [15].
-Radiotherapy alone: it could be considered when there are multiple positive neck nodes (without extranodal extension), perineural invasion, vascular invasion, lymphatic invasion, pT3 or pT4 primary, oral cavity or oropharyngeal primary cancers with positive level IV or V nodes (I-A).
-Concurrent chemorradiotherapy (CCRT): three-weekly intravenous cisplatin 100 mg/m^2^ at days 1, 22, 43) in high-risk pathological features: extracapsular lymph node extension and/or affected margins (I-A) [16, 17]. Weekly 40 mg/m^2^ cisplatin can be non-inferior alternative with better safety profile (IB) [18].

### Specific recommendations in special circumstances

-Oral cavity: in clinically node-negative cases, elective ipsilateral node dissection is recommended more than watchful waiting approach (I-A) [10].
-Unfit patients not candidate for platinum: consider administration of RT alone (I-A). There is no evidence for using agents such as cetuximab or carboplatin in the adjuvant setting [14].

### Radiotherapy-based treatment

**Fig. 1:**
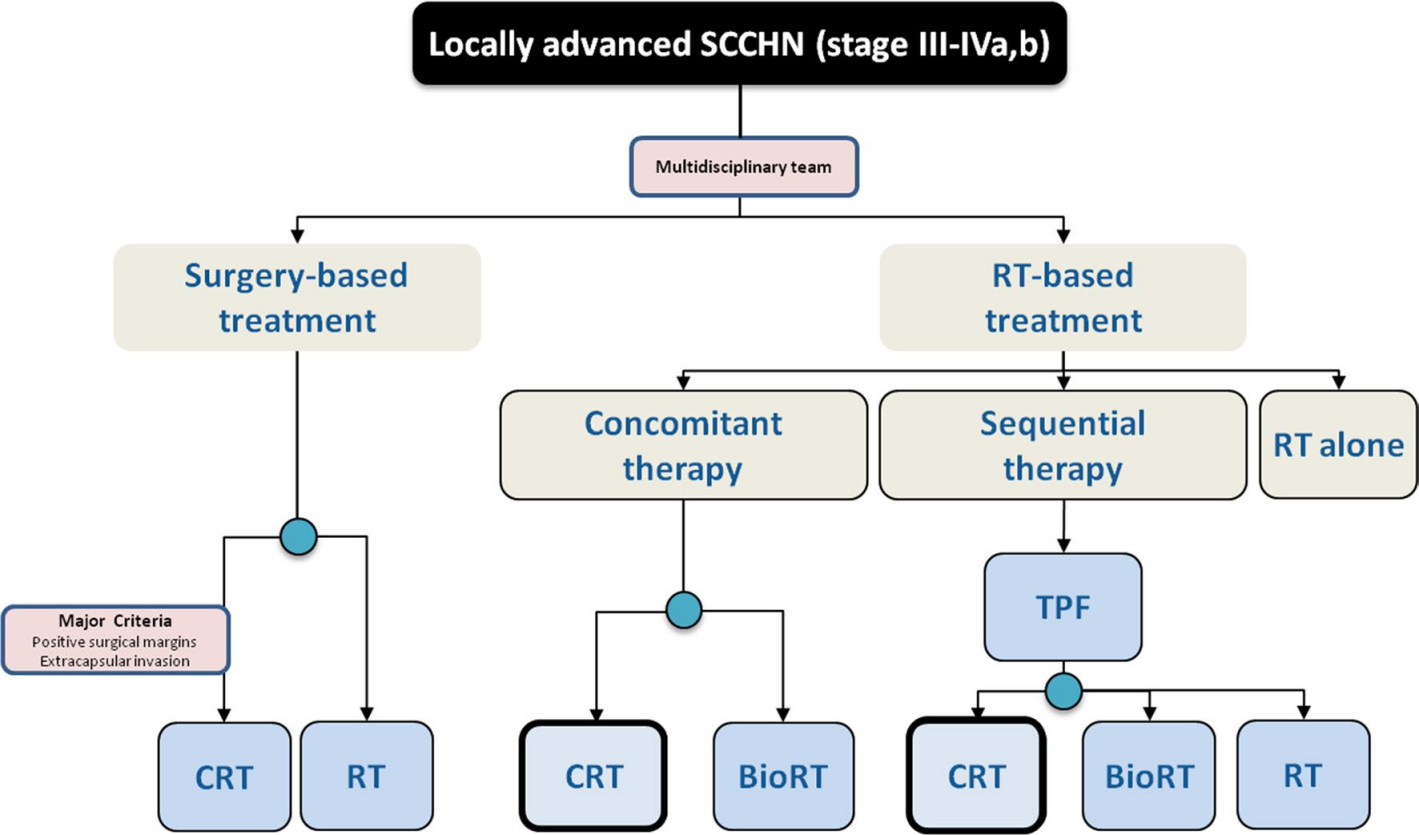
Treatment options in locally advanced SCCHN

CCRT is preferred for patients that are not candidates for or refuse radical surgery (IA) (Fig. 1).

- RT technique: IMRT is preferred (IA) [19]: with similar overall survival compared with conventional RT, it has shown reduction in xerostomia (IA) [15] and probably shorter duration of feeding tube placement (V) [20].
- RT dose: for primary tumor and involved lymph nodes a total of 66 Gy (2.2 Gy/fraction) to 70 Gy (2.0 Gy/fraction) (IA) For elective irradiation of risk sites 44–50 Gy (2.0 Gy/fraction) is proposed (IA) [21–23].
- Chemotherapy regimen: The standard schedule is cisplatin (100 mg/m^2^ days 1, 22, 43) (IA). Weekly cisplatin and other drug combinations have not demonstrated to be equivalent to high-dose cisplatin (IIB) [24, 25]. Concomitant cetuximab is an alternative treatment (400 mg/m^2^ at initial dose day -8 followed by 250 mg/m^2^ weekly concurrent) for patients with some contraindication for cisplatin such as neuropathy, nephropathy, heart disease and hearing loss (IA) [24].

Sequential therapy with induction chemotherapy (ICT) followed by CCRT or RT alone is an option for locally advanced tumors (IIB) (Fig. 1). Factors such as patients' comorbidities, high tumor volume and rapid tumor growth will influence its indication by a multidisciplinary team. ICT has not demonstrated improvement in overall compared with concurrent CRT but increase the response rate. Its limitation is the potential toxicity that could compromise the posterior CRT compliance.

The most recommended induction regimen is TPF schedule (three-weekly Cisplatin 75 mg/m^2^, Docetaxel 75 mg/m^2^, 5-Fluorouracil 750 mg/m^2^/d continue infusion 96 h) (IA) [25].

After ICT, there is no consensus for locoregional treatment (RT, chemoradiotherapy or RT plus cetuximab) (IIB). Decision should be made according to the response and tolerance to previous ICT [26]. Salvage neck dissection should be considered in patients with residual lymph node disease and a complete response in the primary tumor.

### Organ preservation (larynx and hypopharynx)

All patients should have a multidisciplinary evaluation regarding their suitably for a larynx-preservation approach. Organ-preservation surgery, CRT and ICT, all with further surgery reserved for salvage, offer the potential for larynx preservation without compromising overall survival. Selection of a treatment option will depend on patient factors, including age, comorbidities, preferences, socioeconomic factors, local expertise and the availability of appropriate support and rehabilitation services.

For selected patients with extensive T3 or large T4a lesions and/or poor pretreatment laryngeal function, better survival rates and quality of life may be achieved with total laryngectomy rather than with organ-preservation approaches and may be the preferred approach (IA).

CCRT offers a significantly higher chance of larynx preservation than RT alone or ICT followed by RT alone (IA). The best available evidence supports the use of high-dose cisplatin as the drug of choice in this setting [27].

There is insufficient evidence to indicate that survival or larynx-preservation outcomes are improved by the addition of ICT before concurrent treatment [28]. However, in the setting of operable cancer with the goal of larynx preservation, response to ICT serves as a surrogate predictive biomarker for successful organ preservation with subsequent RT plus cisplatin.

**Fig. 2:**
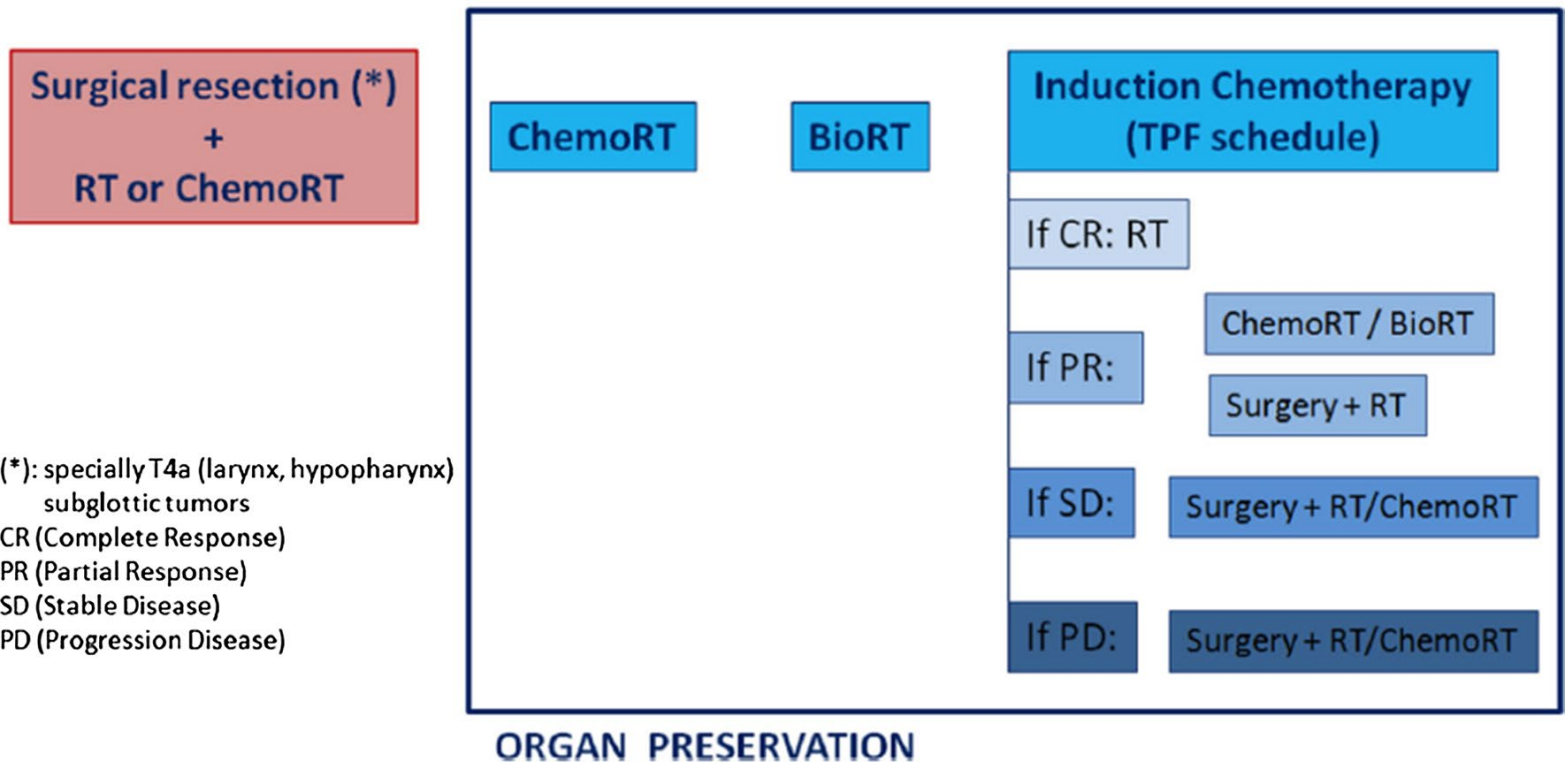
Organ preservation: Larynx/hypopharynx tumors candidate to total laryngectomy

Three options could be considered: Three options could be considered:

- 1.Surgical resection (total versus partial laryngectomy + neck dissection) followed by RT (IA)
  •Specially in T4a (IA).
  •For the most part of subglottic tumors (IA).
    Non-surgical organ preservation alternatives are showed in Fig. 2:
- 2.CRT with three-weekly cisplatin is recommended if patient refuses surgery (IA). If cisplatin cannot be administered, consider cetuximab concurrent to RT (IA).
- 3.ICT with TPF schedule:
  •If complete response of the primary tumors (without lymph node progression) → RT(IA).
  •If partial response (50% reduction of primary tumor without lymph nose progression) → RT (IA) or concomitant RT (with cisplatin or cetuximab) [29] (IIB).
  •If stable disease (primary tumor) or progression → total laryngectomy (including neck dissection) followed by RT (IA) or CRT (IIB) based on histopathological results.

## HPV-related oropharyngeal cancer: a new biological and clinical entity

HPV status has widely been described as an independent predictor of improved outcomes in squamous cell oropharyngeal cancer (OPSCC) patients, proving a 58% reduction in the risk of death for patients with HPV-related OPC as compared HPV-negative tumors [30].

### Diagnosis and staging

p16^INK4a^ over-expression is a surrogate marker of HPV involvement and it is the most widely implemented technique in the clinical setting. Nevertheless, a recent study highly recommends confirming HPV relatedness in p16-positive patients with an HPV specific biomarker such us HPV DNA (IIA) [31]. Double testing for oropharyngeal HPV-related patients is especially important in our geographical area [31, 32].

Importantly, HPV-related oropharyngeal cancer patients’ staging should be done following AJCC TNM 8th edition, whereas clinical decision-making should follow AJCC TNM 7th edition.

### Early disease

Minimally invasive surgery (TORS or TLM) or IMRT monotherapies are both validate techniques for early stages (IA). Importantly patient characteristics and wishes, functional outcomes and expertise of the treating team should be considered.

### Locally advanced (LA) disease

The good prognosis has led the scientific community to develop de-escalation clinical trials for LA HPV-related OPSCC patients [33]. Two phase III de-escalation clinical trials have maintained cisplatin (100 mg/m^2^ every 3 weeks) in combination with RT (70 Gy in 35 fractions) as the standard of care (IA) [34, 35]. Deintensification protocols should be undertaken only within the context of clinical trials.

### Recurrent or metastatic (R/M) disease

Surgery or re-irradiation should always be assessed by the multidisciplinary team for oligometastasic patients. If a radical approach is not possible, the clinical management of R/M HPV-related oropharyngeal patients does not differ from R/M HPV-negative HNSCC, except for patients included on specific clinical trials (IA).

### R/M disease treatment

The multidisciplinary team will assess the benefit of salvage surgery or re-irradiation. In the presence of oligometastatic disease, treatment with curative intent should also be discussed. Systemic treatment will be considered in all other patients. All subjects should be recommended including in clinical trials if available.

### First-line treatment

Decisions will be made based on Eastern Cooperative Oncology Group (ECOG) performance status (PS) comorbidities, symptom burden and PD-L1 expression (in archival or newly tumor samples and characterized by the combined positive score (CPS)).

**Fig. 3:**
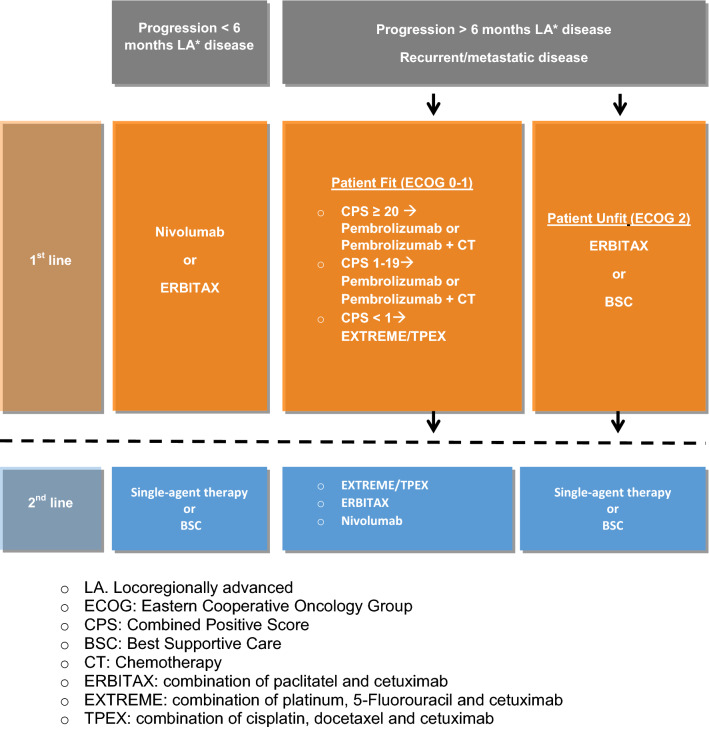
Recurrent/metastatic disease treatment. All patients should be recommended including in clinical trials if available. *LA* Locoregionally advanced, *ECOG* Eastern Cooperative Oncology Group, *CPS* combined positive score, *BSC* best supportive care, *CT* chemotherapy, *ERBITAX* combination of paclitatel and cetuximab, *EXTREME* combination of platinum, 5-Fluorouracil and cetuximab, *TPEX* combination of cisplatin, docetaxel and cetuximab

1. Chemotherapy-naïve patients or patients with progressive disease more than 6 months after locoregional treatment with cisplatin (Fig. 3):
  - Pembrolizumab alone is preferred in patients with PS 0/1, CPS ≥ 20 and low symptom load (IA) [36].
  - The combination of Pembrolizumab plus chemotherapy might be preferred for patients whose symptom burden indicates a greater importance of objective response. (IA) [36].
  - In patients with CPS 1–19 the combination of Pembrolizumab plus chemotherapy (platinum plus 5 FU) is the treatment of choice too (IA) [37].
  - If CPS < 1 or the patient cannot be treated with an immunotherapy protocol EXTREME (combination based on platinum plus 5-FU plus cetuximab) (IA) [38] or TPEX (combination based on cisplatin plus docetaxel plus cetuximab if there is any contraindication to 5-FU) only in PS 0/1 patients able to receive cisplatin (IIB) [39]* are the best option.
  - Best supportive care is the treatment of choice in patients with PS 2. In these patients and those with comorbidities that could not receive platinum the combination ERBITAX (paclitaxel plus cetuximab) should be considered (IIB) [40].
  - The treatment of choice for patients with PS 3/4 is best supportive care.
2. Patients who have received chemotherapy at least 200 mg/m2 of cisplatin for locoregional disease within 6 months after last cisplatin dose should not receive cisplatin or carboplatin. The first option will be Nivolumab (IA) [41] or Pembrolizumab in those patients with a tumor positive score (TPS) >/=50% (IIA) [42]. According to PS or symptom burden treatment with ERBITAX [40] could be an alternative (Fig. 3).

## Second and subsequent line treatment

With the paradigm switch at the frontline we can find new treatment settings:

1. After platinum-based therapy: immunotherapy with Nivolumab (IA) [41] Pembrolizumab in those patients with tumor positive score (TPS) PDL1 ≥ 50% (IIA) [42]
2. After pembrolizumab alone: combination EXTREME is preferred or ERBITAX according to PS (IIIC).
3. After pembrolizumab plus platinum and 5-FU: combination ERBITAX (IIIC).
4. Other cases: considered according to PS single-agent therapy with taxanes (docetaxel, paclitaxel) or anti-metabolite drugs (capecitabine or 5-FU, methotrexate) (IIC). Second and subsequent-line trials tested different drugs with small sample sizes and patient heterogeneity which makes the evaluation of the relative efficacy of each drug challenging. There is no evidence of higher efficacy among the different drugs in the meta-analyses performed [43].

## Supplementary Information

Below is the link to the electronic supplementary material.

Supplementary file1 (DOCX 15 KB)
