# Supplementary material for: SEOM clinical guidelines for the treatment of head and neck cancer (2020)
Source: Clin Transl Oncol. 2021 Feb 26;23(5):913–21. doi: 10.1007/s12094-020-02533-1 (PMC8057973; doi:10.1007/s12094-020-02533-1)
Supplement: Supplementary file 1 — Supplementary file1 (DOCX 15 KB) [file 12094_2020_2533_MOESM1_ESM.docx]

ADDITIONAL TABLE

| **Recommendations** | **Level of evidence** |
| --- | --- |
| **Diagnosis:** PET-CT is recommended in stage III–IV disease when definitive treatment is indicated or in those with equivocal findings on CT or MRI | III,A |
| **Diagnosis:** A biopsy is mandatory. HPV surrogate marker p16 immunohistochemistry in oropharynx and oral cavity tumours is strongly recommended | I,A |
| **Staging:** Accurate staging is crucial to coordinate and tailor therapy to each individual patient. | I,A |
| Cervical lymph nodes treatment based on sentinel node biopsy in T1-2N0 oral and oropharyngeal carcinoma | I,A |
| **Early disease:** If indicated cervical disection en T1-2N0 oral and oropharyngeal carcinoma, elective neck disection preferred over therapeutic neck disetion | I,A |
| **Early disease:** Elective treatmente of the neck in T1-2N0 hypopharynx and supraglòtic càncer is recommended | II,B |
| Early disease: Elective treatment of the neck in T1-2N0 glottic neoplasm is not recommended | III,C |
| **Preferred Radiation technique as adjuvant treatment:** Intensity Modulated Radiotherapy (IMRT) | I,A |
| Preferred CT schedule as adjuvant: Three-weekly intravenous cisplatin 100 mg/m2 at days 1, 22, 43) in high-risk pathological features: extracapsular lymph node extension and/or affected margins | I,A |
| Alternative CT schedule as adjuvant: Weekly 40 mg/m2 cisplatin is a non-inferior alternative with better safety profile* | I,B |
| **Oral cavity Surgery:** in clinically node-negative, elective ipsilateral node dissection is recommended better than watchful waiting approach | I,A |
| **Unfit patients not candidate for platinum in the adjuvant setting:** consider administration of radiotherapy alone. There is no evidence for using alternatively other agents as cetuximab or carboplatin in the adjuvant setting | II,C |
| **Based on RT treatment:** Chemoradiation treatment is standard treatment | I,A |
| **Based on RT treatment:** High dose Cisplatin (100 mg/m2 D1, D21, D42) for concurrent chemoradiotherapy | I,A |
| **Based on RT treatment:** IMRT (intensity-modulated radiation therapy) is preferred for concurrent chemoradiotherapy | I,A |
| **Based on RT treatment:** Induction chemotherapy is an option for high tumor burden and rapidly growing tumors | II,B |
| For selected patients with extensive T3 or large T4a lesions and/or poor pretreatment laryngeal function, better survival rates and quality of life may be achieved with total laryngectomy rather than with organ-preservation approaches and may be the preferred approach | I,A |
| **Organ preservation:** Concurrent chemoradiotherapy (CRT) with cisplatin offers a significantly higher chance of larynx preservation than RT alone or induction chemotherapy followed by RT | I,A |
| **Organ preservation:** If cisplatin cannot be administered: cetuximab concurrent to RT | IIB |
| **Organ preservation:** For selected patients with extensive T3 or large T4a lesions and/or poor pretreatment laryngeal function, better survival rates and quality of life may be achieved with total laryngectomy rather than with organ-preservation approaches and may be the preferred approach | I,A |
| **HPV-related OPC early disease:** Minimally invasive surgery (TORS or TLM) or IMRT monotherapies are both validate techniques | I,A |
| **HPV-related OPC locally-advanced disease**: cisplatin (100 mg/m2 every 3 weeks) in combination with RT (70 Gy in 35 fractions) | I,A |
| **HPV-related OPC R/M disease:** clinical management does not differ from R/M HPV-negative HNSCC, except for patients included on specific clinical trials | I,A |
| **Recurrent/metastatic disease** with progression < 6 months: Nivolumab or ERBITAX | I,A or II,B |
| **Recurrent/metastatic disease** with progression > 6 months and ECOG 0-1. CPS ≥ 20: Pembrolizumab alone or in combination with PF. CPS 1-19: Pembrolizumab in combination with PF and CPS < 1: EXTREME. | I,A |
| **Recurrent/metastatic disease** with progression and ECOG 2: Best supportive care or ERBITAX. | II,B |

* Studies reported as abstracts in congresses pending journal publication at the time of writing this guide.
